# IHACRES, GR4J and MISD-based multi conceptual-machine learning approach for rainfall-runoff modeling

**DOI:** 10.1038/s41598-022-16215-1

**Published:** 2022-07-15

**Authors:** Babak Mohammadi, Mir Jafar Sadegh Safari, Saeed Vazifehkhah

**Affiliations:** 1 Department of Physical Geography and Ecosystem Science, Lund University, Sölvegatan 12, SE-223 62 Lund, Sweden; 2 Department of Civil Engineering, Yaşar University, Izmir, Turkey; 3 Climate Services, World Meteorological Organization, Geneva, Switzerland

**Keywords:** Hydrology, Hydrology

## Abstract

As a complex hydrological problem, rainfall-runoff (RR) modeling is of importance in runoff studies, water supply, irrigation issues, and environmental management. Among the variety of approaches for RR modeling, conceptual approaches use physical concepts and are appropriate methods for representation of the physics of the problem while may fail in competition with their advanced alternatives. Contrarily, machine learning approaches for RR modeling provide high computation ability however, they are based on the data characteristics and the physics of the problem cannot be completely understood. For the sake of overcoming the aforementioned deficiencies, this study coupled conceptual and machine learning approaches to establish a robust and more reliable RR model. To this end, three hydrological process-based models namely: IHACRES, GR4J, and MISD are applied for runoff simulating in a snow-covered basin in Switzerland and then, conceptual models’ outcomes together with more hydro-meteorological variables were incorporated into the model structure to construct multilayer perceptron (MLP) and support vector machine (SVM) models. At the final stage of the modeling procedure, the data fusion machine learning approach was implemented through using the outcomes of MLP and SVM models to develop two evolutionary models of fusion MLP and hybrid MLP-whale optimization algorithm (MLP-WOA). As a result of conceptual models, the IHACRES-based model better simulated the RR process in comparison to the GR4J, and MISD models. The effect of incorporating meteorological variables into the coupled hydrological process-based and machine learning models was also investigated where precipitation, wind speed, relative humidity, temperature and snow depth were added separately to each hydrological model. It is found that incorporating meteorological variables into the hydrological models increased the accuracy of the models in runoff simulation. Three different learning phases were successfully applied in the current study for improving runoff peak simulation accuracy. This study proved that phase one (only hydrological model) has a big error while phase three (coupling hydrological model by machine learning model) gave a minimum error in runoff estimation in a snow-covered catchment. The IHACRES-based MLP-WOA model with *RMSE* of 8.49 m^3^/s improved the performance of the ordinary IHACRES model by a factor of almost 27%. It can be considered as a satisfactory achievement in this study for runoff estimation through applying coupled conceptual-ML hydrological models. Recommended methodology in this study for RR modeling may motivate its application in alternative hydrological problems.

## Introduction

Rapid climate change is causing significant issues over natural resources as well as human beings^1^. Considering water resources management, the accurate estimation of accessible water resources and knowledge about the interactions between the key factors are necessary^2–4^. In this context, runoff gains huge attention which plays a crucial role in estimating the accessible water resources in the future. It is necessary to predict the runoff for the quantity and quality of the available water resources and their management, the design capacity of hydraulic structures, and the associated natural disasters like floods and related environmental issues^5–8^.

Runoff is the main variable for the hydrological analysis from catchment to a continent and global scale which is in direct interaction with rainfall, groundwater, soil moisture, humidity and snow. In addition, various meteorological and climatological variables like temperature, evaporation, humidity, and air pressure provoke the volume of runoff^9^. The nature of hydrological systems could be monitored through the various types of hydrological models which give a deeper insight into the physical interaction between the various parameters and their response to each other^10–13^.

The precise estimation of rainfall-runoff (RR) interactions is a major topic among hydrologists since it can lead managers to have an adequate estimation of the available runoff in the rivers and avoid the negative consequences in the existing hydraulic facilities^14–16^. The heterogeneous pattern of the hydrological components over the basins and their nonlinear behavior make the RR process a complex phenomenon. For this reason, numerous RR models are being developed which aim to increase the precision in predicting the runoff in different spatial and temporal resolutions. Generally, these models could be classified into two fundamental types; the physically-based or white-box and, the machine learning (ML) or the black-box models; where each type comes with different advantages and limitations^11^. For instance, the white-box models require more variables and data (e.g., soil characteristics, topography, land use, etc.) compared to the black-box models which can assist with a few data types. However, the morphological and physical features are presented in different levels with white-box models but are masked and not considered with the black-box models.

In this context, a wide variety of RR models have been employed for modeling runoff in Switzerland. For instance, Antonetti et al.^17^ introduced the revised version of the PREVAH hydrological model which is capable of simulating heavy rainfall events more realistically compared to the traditional version. Antonetti and Zappa^18^ examined the effect of various expert knowledge levels on the accuracy of conceptual hydrological models in Emme catchment, Switzerland. The result augments the better accuracy of more complex models compared to the less expert knowledge. Muelchi et al.^19^ studied the impact of climate change on the runoff regimes in Switzerland using different regional climate models’ precipitation data and a semi-distributed hydrological model. They revealed the runoff decreased in summer and autumn and increased in the winter however, the annual mean was projected to decrease in many catchments of Switzerland. Recently, Rottler et al.^20^ examined the seasonality of flood events in the Rhine river under various climate models’ data for the future. They indicated that the temperature controls the total runoff at the Basel station which is the closest station upstream of the Rhine river. They also showed the change of many snowfalls to the rainfall events which eventually increased the total annual runoff values with the maximum increase in winter.

To solve a variety of environmental problems in the basin, hydrological models can be implemented which are commonly used tools for the design and planning of water resources systems. The distributed white-box hydrological models can display the spatial variation of the process by considering the physics of the problem. On the other hand, black-box models which are also known as empirical approaches are established on the data without considering the physics of the problem^21^. However, due to the complexity of some hydrological modeling such as RR modeling, ML application has attracted the interest of many researchers. The complexity of the RR modeling can be linked to the non-stationary characteristics of the parameters including trend, jump, seasonality, and most importantly non-linearity of the problem. ML approaches can approximate a nonlinear function established on the data to determine a certain relationship among system variables without having information about the physics of the RR process^8,22,23^. It is conducted by using some hydro-meteorological variables and mostly incorporating the observed rainfall and runoff data^24^. As examples from the literature, the outperformance of the wavelet-gene expression programming (W-GEP) model to GEP was documented by Shoaib et al.^25^ using several datasets collected from different regions. The satisfactory performance of an emotional artificial neural network (EANN) for RR modeling in comparison to the artificial neural network (ANN) was presented by Nourani et al.^26^. Chang et al.^27^ applied a self-adaptive fuzzy inference network (SaFIN) for RR modeling in different basins. Nournai et al.^28^ applied the wavelet-M5 model tree for the same purpose and found out that multilinear models may give reliable results for catchments having regular rainfall patterns^29,30^. Tikhamarine et al.^23^ optimized ANN, least squares support vector machine (LSSVM), and multiple linear regression model (MLR) using Harris Hawks Optimization (HHO) and particle swarm optimization (PSO) and showed the higher accuracy of the LSSVM-HHO model for RR modeling. Safari et al.^24^ recommended the regression in the reproducing kernel Hilbert space (RRKHS) approach for RR modeling and demonstrated its accuracy in capturing peak runoff values in contrast to the radial basis function artificial neural network (RBFNN) and multivariate adaptive regression splines (MARS) benchmarks. Morales et al.^31^ introduced a self-identification neuro-fuzzy inference model (SINFIM) for RR modeling in a Chilean watershed where the rainfall and runoff lags and the number of membership functions were determined through the modeling procedure. Better performance of SINFIM was illustrated in comparison to the ANN, adaptive neuro-Fuzzy inference system (ANFIS), and Long Short-Term Memory (LSTM) methods.

The aforementioned studies only investigated the RR process either by with-box or black-box models. Although both approaches have certain advantages where the former gives insights into the physics of the problem and the latter has robust computation ability, their main limitations respectively are lower computation precision and neglecting the physics of the RR process. In this study, in order to overcome the deficiencies of white-box and black-box models, a coupling approach is implemented to consider the physics of the problem through modeling the RR process utilizing white-box hydrological models together with the application of robust ML techniques. We aim to model the RR interaction on a process-based methodology through.

(i) Using several common white-box models.
(ii) Applying the black-box models with some extra variables which were not considered at the previous stage.
(iii) Application of optimization algorithms over the output of the previous stage.
(iv) Introducing a new strategy for improving the ability of ordinary hydrological models in a snow-covered basin.

## Materials and methods

### White-box hydrological models

#### GR4J

The Génie Rural à 4 paramètres Journalier (GR4J) model can be used for hydrological models such as runoff modeling and flood forecasting^32^. This model considers the variables of precipitation, evapotranspiration, and transpiration as flow data for the runoff simulation^33^. If the amount of precipitation (*P*) is more than the amount of evapotranspiration (*E*), then the net precipitation (*P*_*n*_) is equal to:

1 $${P}_{n}=P-E$$

2 $${E}_{n}=0$$

3 $${P}_{S}=\frac{{x}_{1}(1-{(\frac{s}{{x}_{1}})}^{2})\mathrm{tanh}(\frac{{P}_{n}}{{x}_{1}})}{1+\frac{s}{{x}_{1}}\mathrm{tanh}(\frac{{P}_{n}}{{x}_{1}})}$$

where *x*_1_ is the maximum capacity of the soil moisture accounting (mm), *P* the net rainfall (mm), *s* the actual amount of storage and *P*_*s*_ precipitation in the level of *s*. If the amount of precipitation is less than the amount of evapotranspiration then, net evapotranspiration (*E*_*n*_), net precipitation (*P*_*n*_) and, potential evapotranspiration of the storage (*E*_*S*_) as part of the *E*_*n*_ can be calculated as follows

4 $${E}_{n}=E-P$$

5 $${P}_{n}=0$$

6 $$ E_{S} = \frac{{s \cdot \left( {2 - \frac{s}{{x_{1} }}} \right) \cdot {\text{tanh}}\left( {\frac{{E_{n} }}{{x_{1} }}} \right)}}{{1 + \left( {1 - \frac{s}{{x_{1} }}} \right) \cdot {\text{tanh}}\left( {\frac{{E_{n} }}{{x_{1} }}} \right)}} $$

Equations (7)–(11) show the *P*_*erc*_ as the amount of infiltration, *P*_*r*_ is a part of precipitation (routing store) and, it was divided into two parts (*Q*_*1*_ and *Q*_*9*_) also, *P*_*u*_ is the updated level of production store. *Q* constitutes 10% of direct runoff, which is obtained through the hydrograph of unit *H*_*u2*_ with 2 *x*_4_ base time [*x*_4_ is base time in unit hydrograph UH1 (days)]. The *Q*_9_ is another part of 90% of the runoff (delay runoff) which is obtained through the hydrograph of unit *H*_*u1*_ with *X*_4_ base time.

7 $${s}_{u}=s+{P}_{S}-{E}_{S}$$

8 $${P}_{erc}={s}_{u}\left[1-{\left[1+{\left(\frac{4{s}_{u}}{9{x}_{1}}\right)}^{4}\right]}^{-1/4}\right]$$

9 $${P}_{r}={P}_{n}-{P}_{s}+{P}_{erc}$$

10 $${Q}_{1}(i)=0.1\times \sum_{k=1}^{m}H{U}_{2}(k)\times {P}_{r}(i-k+1)$$

11 $${Q}_{9}(i)=0.9\times \sum_{k=1}^{l}H{U}_{1}(k)\times {P}_{r}(i-k+1)$$

Equation (12) shows *F* as groundwater exchange and Eqs. (13)–(16) indicate *R* as the routine moisture storage, *Q* the final runoff, *Q*_*r*_ and *Q*_*d*_ runoff in the outlet and direct runoff, respectively^10^.

12 $$F={x}_{2}{(\frac{R}{{x}_{3}})}^{7/2}$$

13 $$R=\mathrm{max}(0;R+{Q}_{9}+F)$$

14 $${Q}_{r}=R \left[1-{\left[1+{\left(\frac{R}{{x}_{3}}\right)}^{4}\right]}^{-1/4}\right]$$

15 $${Q}_{d}=\mathrm{max}(0;{Q}_{1}+F)$$

16 $$Q={Q}_{r}+{Q}_{d}$$

where *x*_2_ and *x*_3_ are the coefficients of groundwater exchange (mm) and the maximum routing store capacity one day ahead (mm), respectively.

#### IHACRES

The IHACRES^34^ is an integrated conceptual RR model whose main purpose is to describe the hydrological behavior of the basin using the lowest possible parameter^35^. This model requires a time series of precipitation and air temperature variables as model inputs to simulate the flow as well as the observational flow data for the model calibration and the accuracy check. The basis of this model is based on two non-linear reduction models and a linear hydrograph model where the non-linear reduction model converts precipitation into effective rainfall by considering the infiltration and evaporation ratio. For the effective rainfall estimation, the basin moisture index and basin saturation index are calculated for each time step. Equations (17–18) are related to effective rainfall (${u}_{k}$) and *SM* index (${\phi }_{k}$), respectively^36^.

17 $${u}_{k}={[c({\Phi }_{k}-l)]}^{p}\times {r}_{k}$$

18 $${\Phi }_{k}={r}_{k}+(1-\frac{1}{{\tau }_{k}}){\Phi }_{k-1}$$

where *c* is the equilibrium coefficient of rainfall, ${\tau }_{k}$ the drying rate, *l* threshold for *SM* index, *p* the non-linear response terms, and ${r}_{k}$ the observed rainfall. The combination of fast flow (${x}_{k}^{(q)}$) and slow flow (${x}_{k}^{(s)}$) components lead to runoff generation (${x}_{k}$) (*k* shows the time) as follows:

19 $${X}_{k}={X}_{k}^{q}+{X}_{k}^{s}$$

20 $${X}_{k}^{(q)}=-{\alpha }_{q}{X}_{k-1}^{(q)}+{\beta }_{q}{u}_{k}$$

21 $${X}_{k}^{(s)}=-{\alpha }_{s}{X}_{k-1}^{(s)}+{\beta }_{s}{u}_{k}$$

22 $${\tau }_{q}=\frac{-\Delta }{\mathrm{ln}(-{\alpha }_{q})}$$

23 $${\tau }_{s}=\frac{-\Delta }{\mathrm{ln}(-{\alpha }_{s})}$$

24 $${v}_{q}=1-{v}_{s}=1-\frac{{\beta }_{s}}{1+{\alpha }_{s}}$$

where ${\alpha }_{q}$ and ${\beta }_{q}$ are constant time parameters for fast flow, and ${\alpha }_{s}$ and ${\beta }_{s}$ are constant time parameters for slow flow. The $\Delta$ is a time interval, ${\tau }_{q}$ and ${\tau }_{s}$ constant time slides of fast and slow daily currents, respectively; ${v}_{q}$ the ratio of fast flow to total flow ($1-{v}_{s}$), and ${v}_{s}$ the relative volume of slow flow^37^.

#### MISD

The MISD is a semi-distributed and lumped RR model (depending on the implemented type) that was first developed by Brocca et al.^38^ to predict flood events in the Upper Tiber River in central Italy. This model mostly focuses on the SM module which is shown to affect the storage capacity and its associated effect on RR modeling. In this study, we applied the lumped version of MISD with the daily rainfall and air temperature data as inputs at the basin level which simulate the gradual changes of soil water into two independent states. Water exits the first layer by evaporation and transpiration, which is calculated through a linear function between potential evaporation and saturated soil however, the infiltration from the soil surface to the root area is calculated using the non-linear relationship^39^. Three different components cause runoff generation in the MISD model, including surface excess saturation, the second soil layer, and the subsurface runoff components. The first two are collected by the instantaneous geomorphological unit hydrograph (IGUH) and routed to the outlet, while the groundwater runoff is transferred to the outlet by a linear reservoir method. The applied MISD model in this study uses the Curve Number method to investigate losses. The IGUH and linear reservoirs are used to track precipitation in sub-basins and areas that discharge directly into the main waterway, respectively. Finally, routing along the main waterway is estimated through a linear broadcast approach.

### Black-box models

#### Multilayer perceptron (MLP)

The artificial neural network (ANN) has been widely used for modeling and classification in different engineering fields. Recently different types of ANN were implemented for different aims. The multilayer perceptron (MLP) is one of the widespread ANN methods which is successfully applied in water science in many cases^40–42^. The current study used MLP as an ANN model for the modeling aim. There are input layers, an output layer, and hidden layers in the structure of all types of ANN.

Through the MLP modeling process, input variables by some preprocessing are considered as the first stages (input layer). The neuron(s) transfers information from the input layer to the hidden layer (by considering input weight and bias unit), and in the hidden layer, the MLP applies some learning algorithm to the data. Finally, the result transfers to the output layer. The number of weights and bias can be calculated by the summation function which is given as follows:

25 $${u}_{k}=\sum_{(i=1)}^{n}{w}_{ki}{x}_{i}+{b}_{k}$$

where *x*_*i*_ denotes the input variable and *n* shows the number of inputs, *b*_*k*_ is a bias term, and *w*_*ki*_ shows the connection weight. The summation function analysis information is based on the activation function of the MLP model. One of the most common types of MLP activation function is the sigmoid function which is given below:

26 $${f}_{k}=1/(1+{e}^{(-{u}_{k})})$$

The final output of the neuron *k* can therefore be obtained by

27 $${y}_{k}={f}_{k}\left(\sum_{(i=1)}^{n}{w}_{ki}{x}_{i}+{b}_{k}\right)$$

The MLP learning process is based on connecting the various network nodes via optimal weights, and then neurons transfer for output of the above equation to the next step (layer) via selected optimum weights^43^. The Levenberg–Marquardt algorithm was used as a training function, and the number of optimal neurons was selected by a trial and error method. Also, the Logarithm of the sigmoid function and the linear function were used as the activation functions from the input layer to the hidden layer and from the hidden layer to the output layer, respectively.

#### Support vector machine (SVM)

The support vector machine (SVM) is one of the supervised learning methods, which originates from statistical learning theory and is used in classification, pattern recognition, and regression issues^44^. To classify linearly inseparable vectors, various kernel functions can be used for multidimensional cartographers viewed in higher-dimensional spaces, including hierarchical polynomials, radial basis function (RBF), or hyperbolic tangents^44^. The RBF kernel function was used in the current study. This method is one of the new methods that have shown good efficiency in hydrological studies in recent years. The basis of this method is a linear classification of data based on the intended vectors to choose a more reliable margin. One of the important features of this method is that, it simultaneously minimizes the experimental classification error and maximizes the geometric margins. Dibike et al. ^45^ suggested SVM for the first time in hydrological studies by applying SVM for runoff modeling, successfully. It is an efficient training system, which is based on the finite optimization theory and uses the principle of minimizing structural errors and making them optimal^45^. The connection between dependent and independent variables is supposed to be defined by an algebraic function ($f(x)$) plus some noises ($\varepsilon$) in a SVM model.

28 $$f(x)={W}^{T}\times \Phi (x)+b$$

29 $$y=f(x)+noise$$

where *W* and *b* are coefficient vector and constant coefficient, respectively, and they are the components of the SVM function, and ∅ is the kernel function.

#### Whale optimization algorithm (WOA)

The (whale optimization algorithm) WOA is a nature-based optimization algorithm that is inspired by the social behavior of whales in nature introduced by Mirjalili and Lewis^46^. The WOA works with a set of random solutions as a starting step, and their position can be updated in each iteration using the algorithm’s operators. Initially, WOA considers that the best solution is bait, and after the best search agent is identified, other search agents update their location relative to the best search agent. This behavior is described as follows:

30 $$\overrightarrow{X}(t+1)={\overrightarrow{X}}^{*}(t)-\overrightarrow{A}.\left|\overrightarrow{C}.{\overrightarrow{X}}^{*}(t)-\overrightarrow{X}(t)\right|$$

where *t* describes the running iteration, $\overrightarrow{X}$ the condition vectors of the whale, X* the condition vector of the best solution and it can be updated if there exists a better solution. If a better answer is available, then X* must be updated in each iteration. The variables *A* and *C* can be calculated as follows^46^:

31 $$\overrightarrow{A}=2\overrightarrow{a}.\overrightarrow{r}-\overrightarrow{a}$$

32 $$\overrightarrow{C}=2.\overrightarrow{r}$$

where *a* is a constant that decreases linearly from 2 to 0 during iterations (in both exploration and extraction stages) and *r* a random vector at a distance of 0 to 1. The ML models implemented in the MATLAB 2020b environment and the optimal parameters of WOA are listed in Table 1.

**Table 1:** The optimal parameters of WOA.

Quantity	Value
Maximum number of iterations	500
Number of whales	40
The minimum limit for generating unit	2
Total losses	0.3
Total load demand	0.05

### Improving white-box hydrological models via machine learning strategy

The current study recommended the improved RR modeling via hydrological models enhanced by ML approaches. For this aim, three levels were considered for the modeling procedure including level 1: focusing on conceptual runoff modeling via the white-box models (IHACRES, GR4J, and MISD) using daily temperature, precipitation, and evapotranspiration data; Level 2: improving the accuracy of hydrological models via ordinary ML approaches (MLP and SVM) and in level 3: improving runoff modeling outcomes from level 2 was considered as input of MLP model coupled by the WOA (MLP-WOA). The main aim of these processes is to improve the ability of worldwide hydrological models by data-fusion and ML approaches for runoff modeling in a snow-covered basin.

**Figure 1:**
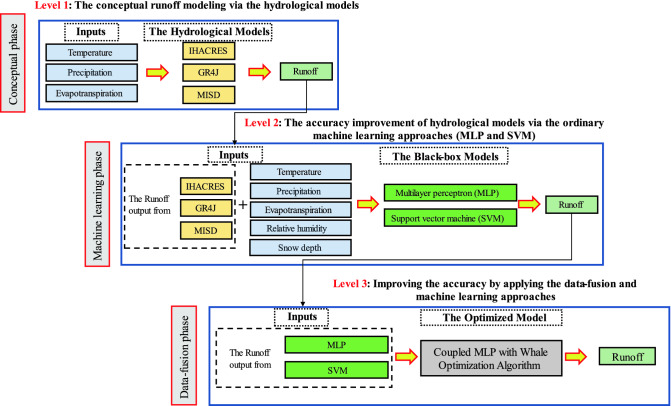
The flowchart of the applied methodology.

The values of the daily runoff time series of the selected study area in Switzerland were firstly simulated through the three white-box hydrological models (IHACRES, GR4J and MISD). For improving the ability of the mentioned classical hydrological model, we applied the ML approaches (MLP and SVM). In the final step of the modeling process, we used a nature optimization algorithm for boosting the ability of hydrological and ML models in runoff simulation. The hybrid nature-inspired model was then proposed to improve the daily runoff simulation using the hybridization of classical MLP with WOA (MLP-WOA). As shown in Fig. 1, the current study combined white-box and black-box approaches via three separated levels as follows; Level 1: temperature, precipitation, and evapotranspiration parameters were considered as inputs of GR4J, IHACRES, and MISD models and the output is simulated runoff via white-box models. Level 2: simulated runoff by GR4J (from level 1), simulated runoff by IHACRES (from level 1), simulated runoff by MISD (from level 1), temperature, precipitation, evapotranspiration, relative humidity, and snow depth were considered as inputs of MLP and SVM models via various scenarios, and the output of level 2 is simulated runoff via black-box models. Level 3: the best simulated runoff by MLP (MLP5 from level 2) and the best simulated runoff by SVM at level 2 were considered as inputs of MLP and MLP-WOA models, and the output of level 3 is simulated runoff via data-fusion tasks. The flowchart of the applied methodology is shown in Fig. 1.

#### Evaluation criteria

The data used in this study is divided into calibration and validation. Among the entire data, 70% and 30% of data were considered for calibrating and validating models, respectively for running white-box and black-box models. The calibration phase was selected from 1 January 1981 to 31 December 2010, and the validation phase was selected from 1 January 2011 to 26 March 2021. Numerous statistical metrics were used for the evaluation of RR models. In this study, the mean absolute error (*MAE*), root mean square error (*RMSE*) and Pearson correlation coefficient (*r*) are used as statistical metrics. In addition, the Nash–Sutcliffe efficiency (*NSE*^47^) and Kling-Gupta efficiency (*KGE*^48^) were utilized, which are based on the goodness-of-fit approach and are among the most common metrics in the hydrological model's evaluation. The aforementioned evaluation criteria can be computed as follows

33 $$MAE=\frac{1}{N}\sum_{i=1}^{N}(|{S}_{i}-{O}_{i}|)$$

34 $$RMSE=\sqrt{\frac{1}{N}\sum_{i=1}^{N}{({S}_{i}-{O}_{i})}^{2}}$$

35 $$r=\frac{\sum_{i=1}^{n}({O}_{i}-\overline{O }).({S}_{i}-\overline{S })}{\sqrt{\sum_{i=1}^{n}{({O}_{i}-\overline{O })}^{2}.\sum_{i=1}^{n}{({S}_{i}-\overline{S })}^{2}}}$$

36 $$NSE=1-\frac{\sum_{i=1}^{n}{({S}_{i}-{O}_{i})}^{2}}{\sum_{i=1}^{n}{({O}_{i}-\overline{O })}^{2}}$$

37 $$KGE=1-\sqrt{{(r-1)}^{2}+{(\frac{{\mu }_{s}}{{\mu }_{o}}-1)}^{2}+{(\frac{C{V}_{s}}{C{V}_{o}}-1)}^{2}}$$

where ${S}_{i}$ and ${O}_{i}$ ($\overline{S }$ and $\overline{O }$) denote simulated and observed (mean) daily runoff, respectively, and *N* the number of observed values used to train and test the models, separately, ${CV}_{o}$ (${CV}_{s}$) the coefficient of variation for observed (simulated) values of the daily runoff. The *r* is the correlation coefficient between observed and simulated values of the daily runoff, ${\mu }_{o}$ the standard deviation of observed daily runoff values, and ${\mu }_{s}$ the standard deviation of simulated daily runoff values.

### The study area and data

**Figure 2:**
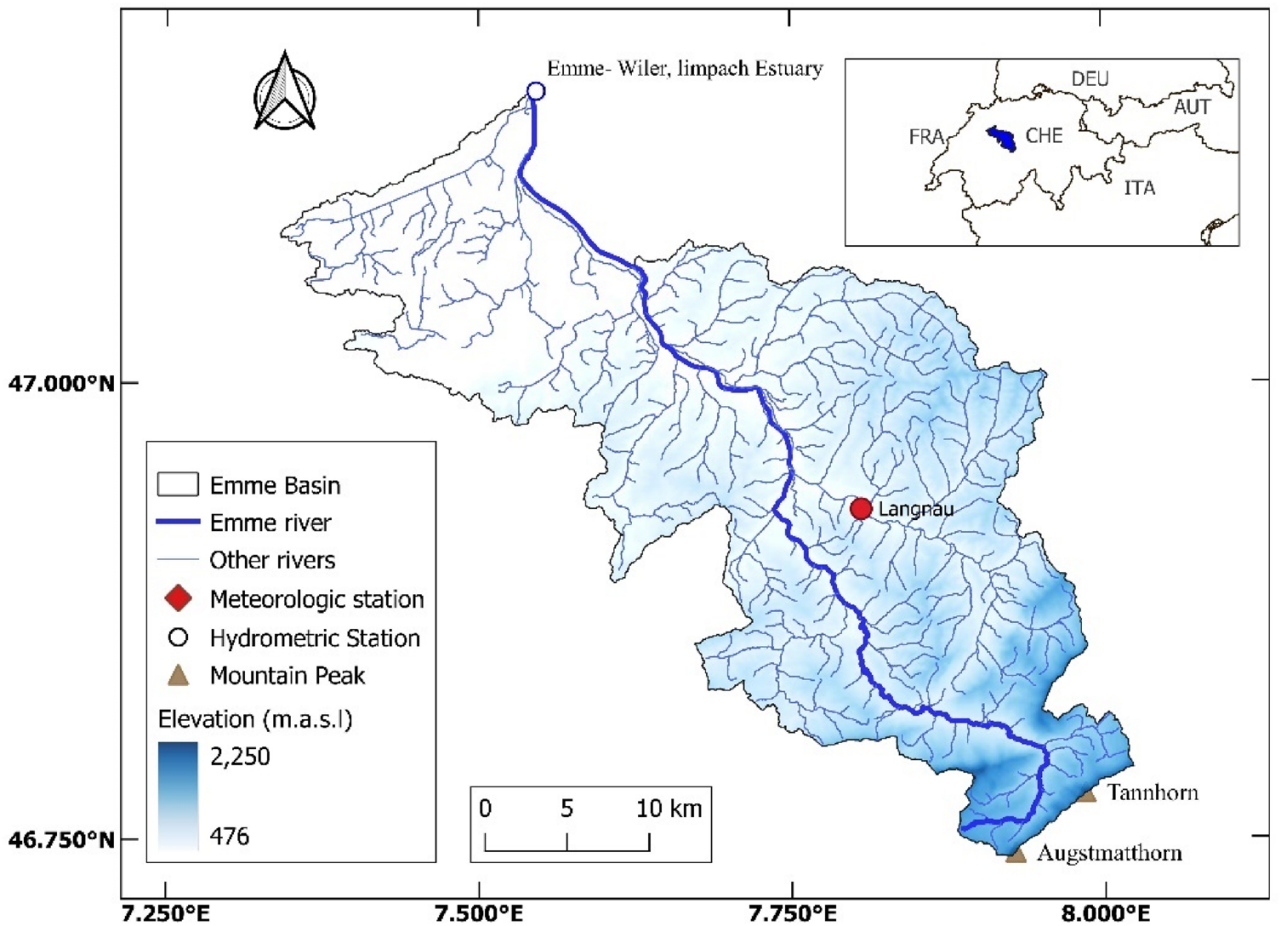
The geographical map of the Emme watershed in central Switzerland. Figure was created using the open-source and free QGIS V3.18. The open-access European Digital Elevation Model (EU-DEM), version 1.1 file was retrieved from Copernicus Land Monitoring Service (https://land.copernicus.eu/imagery-in-situ/eu-dem/eu-dem-v1.1?tab=metadata). The catchment shape file and the river network geospatial data were retrieved from the open-access Hydrological Atlas of Switzerland (https://hydromaps.ch/)^49^.

The Emme catchment (shown in Fig. 2) was chosen as a pilot study area which is located in central Switzerland mainly in the Canton of Bern with an approximate area of 924 km^2^. The mountainous Pre-Alps region with 2150 m height around the Augstmatthorn and Tannhorn peaks is the source of 82 km Emme river which drains to the Aare river near the city of Solothurn at 430 m that eventually drains the Rhine river. The catchment’s mean altitude is around 860 m.

**Figure 3:**
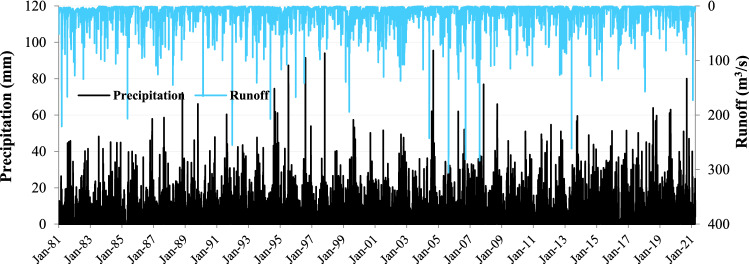
The plot of the applied observed precipitation and runoff time series.

The daily precipitation (*P*), mean, minimum and maximum temperature (*T*), relative humidity (*RH*), evapotranspiration (*ET*), and snow (*S*) data for the period January 1974–March 2021 are obtained from the MeteoSwiss for the Langnau station which is located around the central regions of the basin. The daily runoff (*Q*) measurement for the same period was gathered for the Emme, Wiler Limpach Estuary hydrometric station from the Swiss Federal Office for the Environment (FOEN). The brief geographic and statistical details of the applied data are presented in Table 2, and the time series of the precipitation and runoff data are shown in Fig. 3. Dataset is categorized into the warm-up section (7 years: 1st January 1974 to 31st December 1980), calibration section (30 years: 1st January 1981 to 31st December 2010), and validation section 10-years: 1st January 2011 to 26th March 2021) for implementing conceptual RR models.

**Table 2:** The statistical characteristics of the applied data.

Variable/Stat	Mean	Min	Max	Med	Std. Dev	Kurtosis	Skewness	Sample size
Precipitation (mm)	3.74	0	95.5	0	7.53	18.07	3.49	17,252
Temperature (°c)	8.15	− 19.8	26	8.4	7.42	− 0.77	− 0.12	17,252
Relative Humidity (%)	81.75	19.6	100	83.6	11.06	− 0.03	− 0.63	17,252
Evapotranspiration (mm)	2.28	0.05	14.45	2.19	0.74	8.13	1.41	17,252
Snow depth (m)	0.34	0	42	0	1.88	92.8	8.4	17,252
Runoff (m^3^/s)	9.51	0.54	305.72	3.67	16.55	50.05	5.42	17,252

## Results

### Conceptual rainfall-runoff modeling using the white-box models

#### Calibration processes

The dataset is categorized into warm-up Sect. (7 years: 1st January 1974 to 31st December 1980), calibration Sect. (30 years: 1st January 1981 to 31st December 2010), and validation Sect. (10 years: 1st January 2011 to 26th March 2021) for implementing of conceptual RR models^50^. For this aim, a numerical optimization method of derivative-free search method (Pattern Search) was used for calibrating the GR4J model. The optimal parameters of the GR4J model are given in Table 3.

**Table 3:** Parameters setting by pattern search approach for GR4J model.

Parameters	Description	Optimal value
*X*1	Maximum capacity of the production store (mm)	24
*X*2	Groundwater exchange coefficient (mm)	− 24.32
*X*3	One day ahead maximum capacity of the routing store (mm)	120
*X*4	The time base of unit hydrograph UH1 (days)	1.34

A modified SCE-UA (shuffled complex evolution method developed at The University of Arizona), as the global optimization technique was employed for calibrating IHACRES conceptual model^51,52^. The optimal values by the SCE-UA method for calibrating the IHACRES model are provided in Table 4. For calibrating the MISD model a trial-and-error method based on expecting all parameters to be in a monotonic space, and according to the Kling Gupta Efficiency was implemented^38^. The MISD calibration results are listed in Table 5.

**Table 4:** Parameters setting by SCE-UA approach for IHACRES model.

Parameters	Description	Optimal value	Typical range
*τs*	Time constant governing rate of recession of slow-flow (day)	59.27	10–350
*τq*	Time constant governing the rate of recession of quick flow (day)	2.95	0.5–10
*d*	Flow threshold (mm)	78.58	50–550
*Vs*	The proportion of slow flow to total flow (proportion)	0.78	0–1
*f*	Plant stress threshold factor (dimensionless)	0.37	0.01–3
*e*	Temperature to potential evapotranspiration conversion factor (dimensionless)	0.25	0.01–1.5

**Table 5:** Parameters setting for implementing MISD model.

Parameters	Description	Considered range	Optimal value
*W_max*	Fixed water capacity 1st layer	150	150
*W_max2*	Total water capacity of 2nd layer	100–3000	935.50
*W_p*	Initial conditions (fraction of W_max)	0.1–0.9	0.1
*m2*	The exponent of drainage for 1st layer	2–1.0	6.75
*Ks*	Hydraulic conductivity for 1st layer	0.1–40	4.30
*gamma1*	Coefficient lag-time relationship	0.5–3.5	1.71
*Kc*	Parameter of potential evapotranspiration	0.4–3	2.99
*alpha*	Exponent runoff	0.1–15	2.48
*Cm*	Snow module parameter degree-day	0.1/24–3	2.29
*m22*	An exponent of drainage for 2nd layer	5–3.5	26.24
*Ks2*	Hydraulic conductivity for 2nd layer	0.01–65	24.09

#### Conceptual rainfall-runoff models evaluation

The results of applied metrics over the white-box models are shown in Table 6. Not surprisingly, the acquired values differ from the applied models where in general, the GR4J shows better performance. The minimum difference between the calibration and validation phase is related to the GR4J model by *RMSE* of 0.4 m^3^/s, and the maximum difference is related to the IHACRES model by *RMSE* of 1.15 m^3^/s. In general, the *NSE* and *KGE* outcomes explain the acceptable performance of the applied models. Although it differs in the calibration and validation phases overall, the GR4J acquires the least *MAE* and *RMSE* as well as the highest for the *NSE*, *KGE*, and *r*. Considering the plot for the measured and simulated, the GR4J and MISD models show better performance over the peak values compared to the IHACRES.

**Table 6:** The results of the applied metrics over different white-box models.

Model	Phase	*MAE*	*RMSE*	*NSE*	*r*	*KGE*
IHACRES	Calibration	6.53	12.76	0.44	0.66	0.527
	Validation	5.83	11.61	0.41	0.64	0.489
GR4J	Calibration	6.11	11.9	0.51	0.74	0.570
	Validation	5.72	11.5	0.42	0.68	0.466
MISD	Calibration	6.44	12.83	0.43	0.7	0.689
	Validation	5.93	11.85	0.39	0.64	0.584

**Figure 4:**
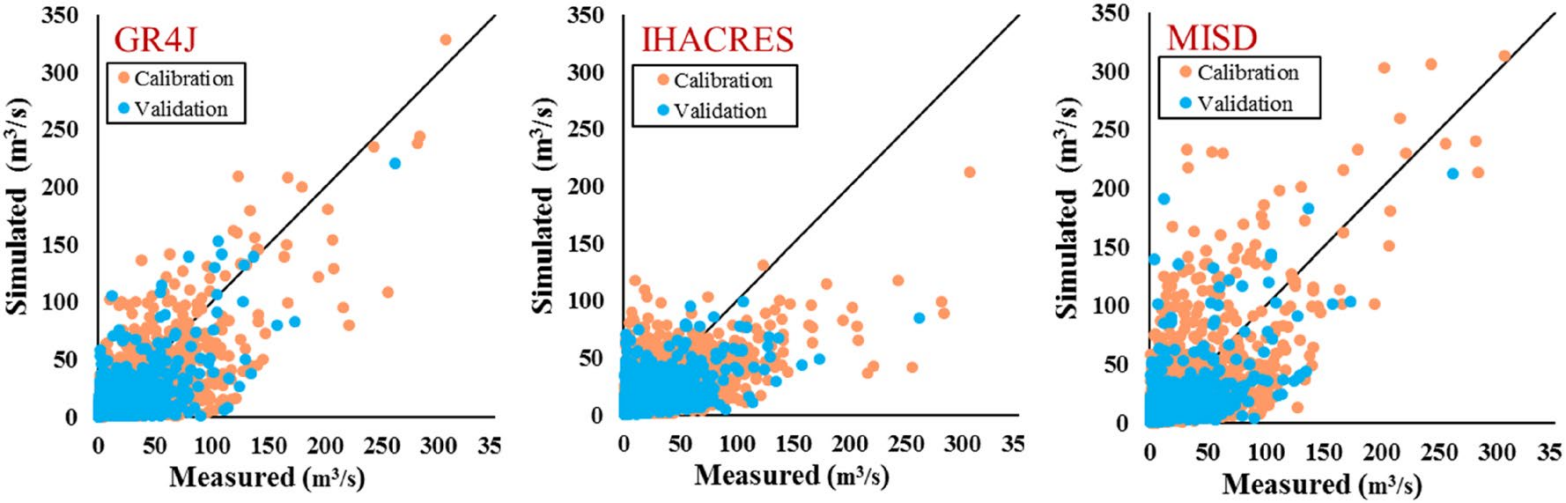
Scatter plots of measured vs simulated runoff using the GR4J, IHACRES and MISD.

The scatter plots of the measured vs simulated runoff using the applied white-box models on their calibration and validation stages are illustrated in Fig. 4. It is shown that IHACRES underestimates the simulation for higher values (over 100 m^3^/s) whereas GR4J captures the higher values with lower deviations. For the same category (less than 100 m^3^/s), several overestimations are derived from the MISD simulations however, the higher values are captured much better.

Figure 4 illustrates that the GR4J model performed the best capability during the calibration (*RMSE* = 11.9 m^3^/s and *r* = 0.74) and validation (*RMSE* = 11.5 m^3^/s and *r* = 0.68) phases. There can be a potential for enhancing the GR4J model ability if we match a proportional fraction of the soil moisture however, this hypothesis requires to be investigated in future research. The fraction of soil moisture in the GR4J model is considered the difference between the available soil moisture and the field capacity. Indeed, in nature, soil moisture obtains from saturation level, and this condition occurs between 2 to 4 days, then soil moisture obtains its field capacity after the drainage process of the soil water. Whereas the GR4J model does not require the upper limit of soil moisture as saturation soil moisture, which can be another reason for the capability of the GR4J model in RR simulation.

**Figure 5:**
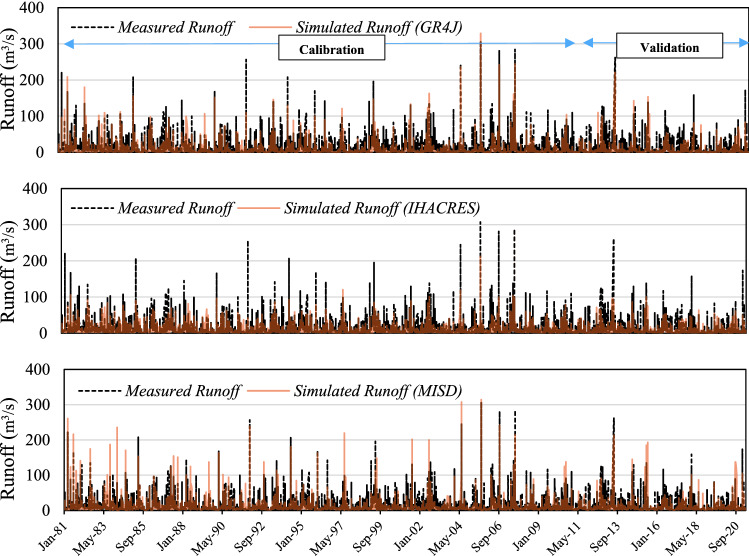
The plot of measured vs simulated runoff time series using GR4J, IHACRES, and MISD models.

The measured hydrograph in the outlet of the catchment and simulated hydrograph by the hydrological models are shown in Fig. 5. Calibration and validation phases were selected according to the time series goals It shows all white-box models have unsatisfactory results in runoff simulation at snow-covered catchments. Although GR4J and IHACRES models detect some extreme values, the MISD provides poor results in both calibration and validation phases.

### Runoff simulation using coupled hydrological models via machine learning approaches

#### Scenario definition

The results of applied metrics for conceptual models were not in a satisfactory domain, so it was decided to improve the accuracy of the acquired output from RR models by applying two widespread ML methods of MLP and SVM, separately. The various scenarios (explained in Table 7) were proposed by applying the ML methods using the runoff output obtained by white-box models in the previous stage coupled with different extra variables^53^.

**Table 7:** Intended scenarios for the implementation of coupled scenarios (machine learning via hydrological models).

No.	Inputs		Models (machine learning)		Output
	The input from phase 1	Meteorological variables			
1	The output runoff from IHACRES	*P*	MLP1	SVM1	Runoff
2		*P* + *T*	MLP2	SVM2	Runoff
3		*P* + *T* + *ET*	MLP3	SVM3	Runoff
4		*P* + *T* + *ET* + *RH*	MLP4	SVM4	Runoff
5		*P* + *T* + *ET* + *RH* + *S*	MLP5	SVM5	Runoff
6	The output runoff from GR4J	*P*	MLP6	SVM6	Runoff
7		*P* + *T*	MLP7	SVM7	Runoff
8		*P* + *T* + *ET*	MLP8	SVM8	Runoff
9		*P* + *T* + *ET* + *RH*	MLP9	SVM9	Runoff
10		*P* + *T* + *ET* + *RH* + *S*	MLP10	SVM10	Runoff
11	The output runoff from MISD	*P*	MLP11	SVM11	Runoff
12		*P* + *T*	MLP12	SVM12	Runoff
13		*P* + *T* + *ET*	MLP13	SVM13	Runoff
14		*P* + *T* + *ET* + *RH*	MLP14	SVM14	Runoff
15		*P* + *T* + *ET* + *RH* + *S*	MLP15	SVM15	Runoff

#### Rainfall-runoff modeling via black-box models

According to Table 7, the current study considered combining the output of each hydrological model via each meteorological variable separately as input of ML models. To this end, based on the scenario defined, the simulated runoff by IHACRES and precipitation were considered as input of ML models (MLP1 and SVM1); the simulated runoff by MISD, precipitation, temperature, evapotranspiration, relative humidity, and snow depth was considered as input of ML models (MLP15 and SVM15), and so on. Based on the results, adding meteorological variables helped all hydrological models for having a better runoff simulation. Adding precipitation, temperature, evapotranspiration, relative humidity, and snow depth increased the ability of the IHACRES model. Therefore, the fifth scenario (MLP5) boosted ability of IHACRES model in runoff simulation by *MAE* = 4.94 (m^3^/s), *RMSE* = 9.43 (m^3^/s), *NSE* = 0.61, and *KGE* = 0.61 in validation phase (according to Table 8).

**Table 8:** The results of the applied metrics on the calibration and validation phases through different MLP scenarios.

Model	Phase	*MAE*	*RMSE*	*NSE*	*r*	*KGE*
MLP1	Calibration	6.28	11.78	0.52	0.72	0.59
	Validation	5.69	10.91	0.48	0.69	0.54
MLP2	Calibration	5.59	10.74	0.6	0.78	0.68
	Validation	5.09	9.85	0.58	0.77	0.58
MLP3	Calibration	5.56	10.47	0.62	0.79	0.69
	Validation	4.99	9.52	0.6	0.79	0.60
MLP4	Calibration	5.56	10.45	0.62	0.79	0.68
	Validation	5.02	9.54	0.6	0.78	0.61
MLP5	Calibration	5.53	10.32	0.63	0.79	0.70
	Validation	4.94	9.43	0.61	0.79	0.61
MLP6	Calibration	6.35	11.36	0.55	0.74	0.63
	Validation	6.07	10.82	0.49	0.7	0.56
MLP7	Calibration	5.63	10.29	0.63	0.8	0.70
	Validation	5.31	9.86	0.58	0.76	0.59
MLP8	Calibration	5.53	10.13	0.64	0.8	0.72
	Validation	5.16	9.65	0.59	0.77	0.62
MLP9	Calibration	5.54	10	0.65	0.81	0.71
	Validation	5.21	9.65	0.59	0.77	0.61
MLP10	Calibration	5.65	10.13	0.64	0.8	0.71
	Validation	5.28	9.61	0.6	0.78	0.62
MLP11	Calibration	6.3	11.41	0.55	0.74	0.62
	Validation	5.84	10.82	0.49	0.7	0.53
MLP12	Calibration	5.82	10.58	0.61	0.78	0.68
	Validation	5.43	10.26	0.54	0.74	0.57
MLP13	Calibration	5.78	10.57	0.61	0.78	0.69
	Validation	5.46	10.23	0.54	0.74	0.58
MLP14	Calibration	5.77	10.64	0.61	0.78	0.68
	Validation	5.38	10.24	0.54	0.74	0.57
MLP15	Calibration	5.73	10.32	0.63	0.79	0.70
	Validation	5.42	10.25	0.54	0.74	0.57

**Figure 6:**
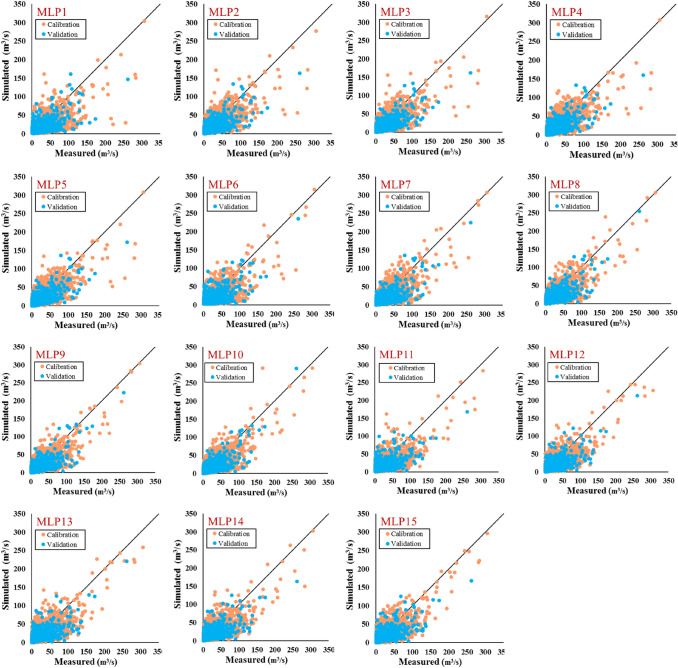
Scatter plots of measured vs simulated runoff for the calibration and validation phases through the different MLP scenarios.

Adding precipitation, temperature, and evapotranspiration were the most useful variables for enhancing the ability of the GR4J model in runoff simulation; such a way that runoff was simulated by *MAE* = 5.16 (m^3^/s), *RMSE* = 9.65 (m^3^/s), *NSE* = 0.59, and *KGE* = 0.62 under the frame of MLP8 at the validation phase. Performance of MISD model was improved by applying precipitation, temperature, evapotranspiration, and relative humidity to MLP model (MLP14); which simulated amount of runoff by *MAE* = 5.38 (m^3^/s), *RMSE* = 10.24 (m^3^/s), *NSE* = 0.54, and *KGE* = 0.57 in validation phase. Figure 6 exhibits the scatter plots of measured vs simulated runoff for the calibration and validation phases through the effect of adding meteorological variables to the hydrological models (based on the MLP model).

**Figure 7:**
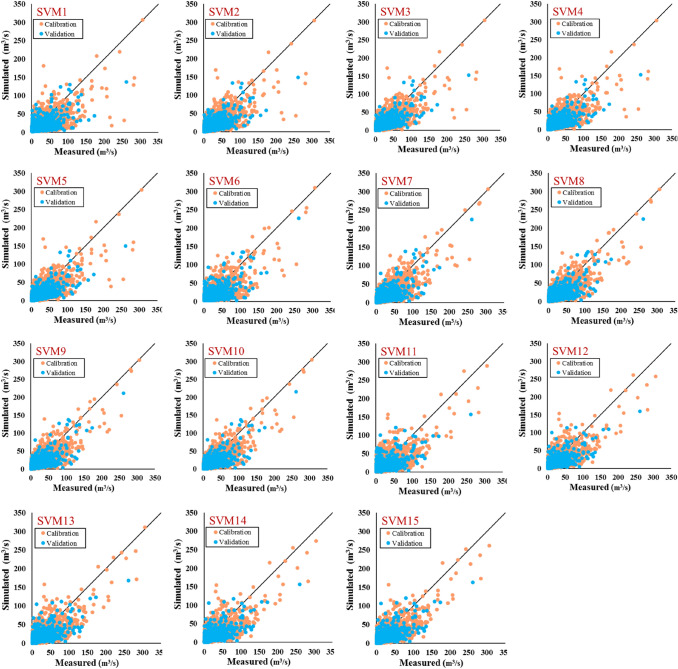
Scatter plots of measured vs simulated runoff of the calibration and validation phases through the different SVM scenarios.

The ability of the SVM model is evaluated as a boosting tool for combining each meteorological variable with hydrological models. As shown in Table 9 and Fig. 7, the best performance is related to combining precipitation, temperature, evapotranspiration, relative humidity, and snow depth by the IHACRES model (SVM5). The SVM scenarios simulated runoff via *MAE* = 5.62 and 5.02 (m^3^/s) and, *r* = 0.78 and 0.77 for calibration and validation phases, respectively. Application of GR4J proved combining precipitation, temperature, and evapotranspiration by this model can have more accurate result in runoff simulation by *MAE* = 5.31 (m^3^/s), *RMSE* = 9.82 (m^3^/s), *NSE* = 0.58, and *KGE* = 0.60 in validation phase. Adding precipitation, temperature, evapotranspiration, relative humidity, and snow depth (SVM15) to the MISD model can make an more accurate approach for runoff simulating by result of *MAE* = 5.50 (m^3^/s), *RMSE* = 10.32 (m^3^/s), *NSE* = 0.53, and *KGE* = 0.59 in validation phase.

**Table 9:** The results of the applied metrics on the calibration and validation phases through different SVM scenarios.

Model	Phase	*MAE*	*RMSE*	*NSE*	*r*	*KGE*
SVM1	Calibration	6.33	11.86	0.51	0.72	0.58
	Validation	5.79	10.93	0.48	0.69	0.53
SVM2	Calibration	5.8	10.96	0.58	0.76	0.65
	Validation	5.23	10.17	0.55	0.75	0.56
SVM3	Calibration	5.68	10.70	0.60	0.78	0.67
	Validation	5.07	9.79	0.58	0.77	0.58
SVM4	Calibration	5.66	10.63	0.61	0.78	0.67
	Validation	5.04	9.82	0.58	0.77	0.57
SVM5	Calibration	5.65	10.57	0.61	0.78	0.68
	Validation	5.02	9.79	0.58	0.77	0.57
SVM6	Calibration	6.32	11.33	0.56	0.75	0.62
	Validation	6.08	10.87	0.48	0.70	0.56
SVM7	Calibration	5.74	10.41	0.62	0.79	0.69
	Validation	5.42	10.13	0.55	0.75	0.59
SVM8	Calibration	5.67	10.2	0.64	0.80	0.70
	Validation	5.31	9.82	0.58	0.77	0.60
SVM9	Calibration	5.66	10.16	0.64	0.80	0.70
	Validation	5.31	9.85	0.58	0.76	0.60
SVM10	Calibration	5.64	10.08	0.65	0.81	0.71
	Validation	5.32	9.84	0.58	0.76	0.60
SVM11	Calibration	6.33	11.45	0.55	0.74	0.62
	Validation	5.93	10.97	0.47	0.69	0.53
SVM12	Calibration	5.98	10.88	0.59	0.77	0.66
	Validation	5.57	10.44	0.52	0.73	0.55
SVM13	Calibration	5.96	10.79	0.60	0.77	0.67
	Validation	5.57	10.38	0.53	0.73	0.56
SVM14	Calibration	5.91	10.75	0.60	0.77	0.66
	Validation	5.51	10.34	0.53	0.73	0.56
SVM15	Calibration	5.82	10.54	0.62	0.79	0.67
	Validation	5.50	10.32	0.53	0.73	0.59

**Figure 8:**
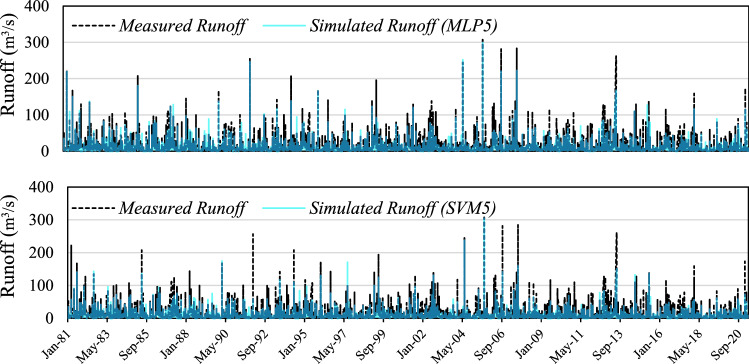
The plot of measured vs simulated runoff time series using MLP5 and SVM5 models.

The measured and simulated hydrographs by best-proposed models including combining IHACRES models via meteorological variables reproduced by MLP and SVM models are shown in Fig. 8. Both applied ML techniques (MLP and SVM) proved adding meteorological variables in a parallel situation can increase the performance of hydrological models in snow-covered catchments. However, there are divergences between the ability of MLP5 a and SMV5 for adding meteorological variables to IHACRES models. As the simulated hydrograph (Fig. 8) shows, MLP5 can have a better simulation of the peak flow (maximum events) in comparison with SVM5. Also, both MLP5 and SVM5 can be nominated as capable tools for adding extra meteorological variables to hydrological process-based models.

### The accuracy improvement by coupled MLP-WOA model

Although the accuracy of the proposed scenarios is slightly better than the RR models implemented in the first stage, it was decided to enumerate a data-fusion model to see whether the accuracy improves. To this aim, the MLP5 and SVM5 which showed the highest performances were selected as inputs of the model in the third phase. Then, the derived runoff from the mentioned models was chosen as input for the application of MLP and MLP-WOA models. In the third phase, firstly simulated runoff by MLP5 and SVM5 were considered as input of ordinary MLP model, which the result of MLP showed the application of the data-fusion approach can improve the accuracy of ordinary MLP and SVM. Then, the WOA optimizer approach was applied for improving the ability of MLP’s training. The outputs of MLP5 and SVM5 are considered as the inputs of the MLP-WOA model. For this aim, the third phase (according to Table 10) benefits from the advantages of physically-based models in the first phase, and advantages of the ML process in the second phase and the advantages of the bio-inspired optimization algorithm in the third phase.

**Table 10:** Intended scenarios for the implementation of MLP and MLP-WOA models.

No.	Inputs	Output	Models	
1	MLP5 + SVM5	*Q*	MLP	MLP-WOA

**Figure 9:**
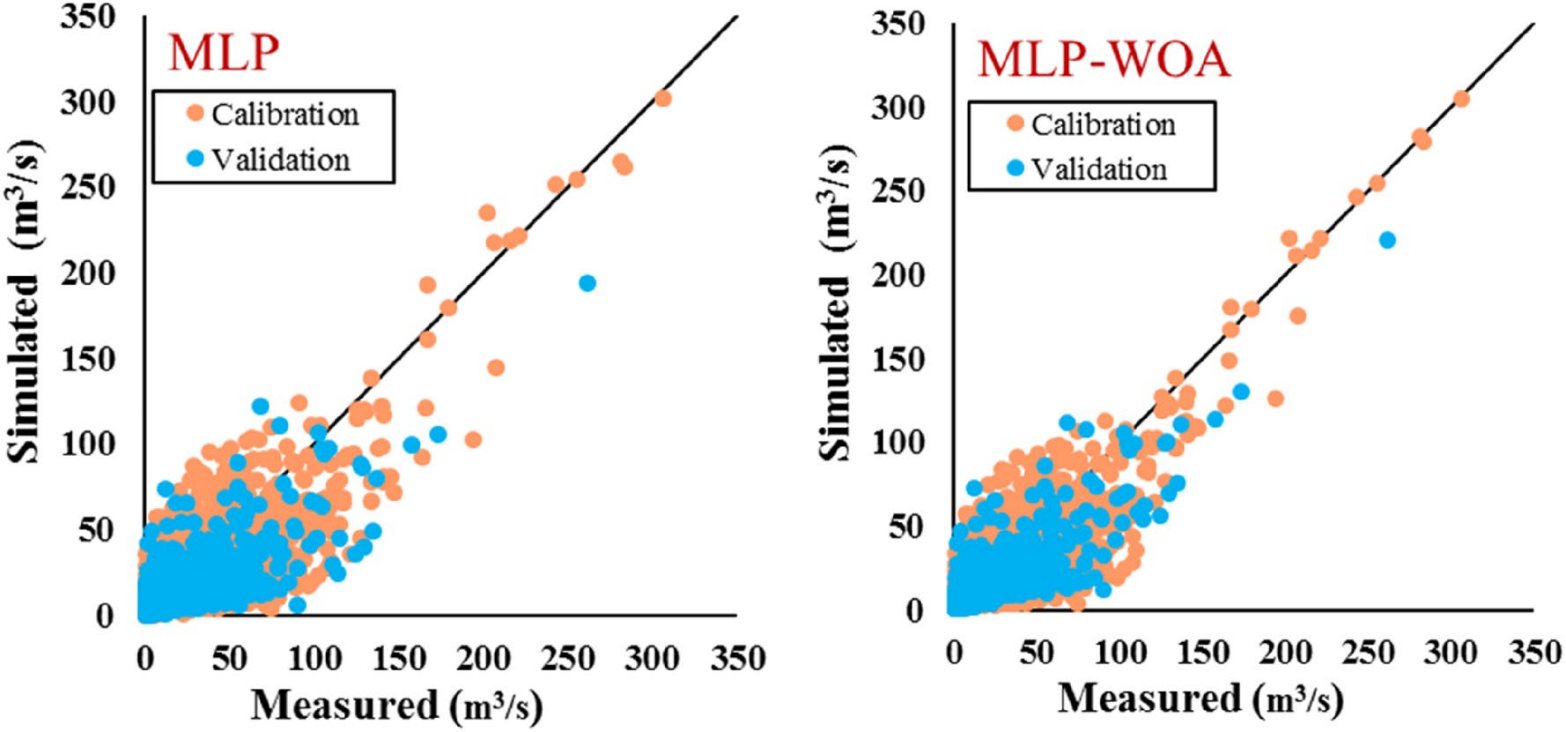
Scatter plots of measured vs simulated runoff on the calibration and validation phases through the MLP and coupled MLP-WOA models.

Results of the third learning phase of the current study are provided in Table 11 and Fig. 9. The main goal of the third stage is the application of an advanced method for coupling the best result of the previous stage (MLP5 and SVM5) to reach a high accuracy in runoff simulation. Then, WOA coupled with MLP (namely MLP-WOA) was considered as an advanced approach for this aim. Two aims were fulfilled in this stage: (*i*) simulated runoff via MLP5 and SVM5 were considered as inputs of the model in the third stage. In this way, the final model can benefit from sages 1 and 2, which means, the final model of the third stage (MLP-WOA) has advantages of black-box and white-box models at the same time. (*ii*) for reaching maximum efficiency, this stage employed a high-performance predictor tool by combining a nature-inspired optimization algorithm via an ordinary ML. Therefore, for the evaluation of the mentioned combined model (MLP-WOA), its performance is evaluated by standalone MLP. The MLP-WOA simulated runoff by result of *MAE* = 5.14 (m^3^/s), *RMSE* = 9.07 (m^3^/s), *NSE* = 0.71, *r* = 0.85, and *KGE* = 0.78 in training phase, and *MAE* = 4.56 (m^3^/s), *RMSE* = 8.49 (m^3^/s), *NSE* = 0.68, *r* = 0.84, and *KGE* = 0.66 in testing period. In addition, the evaluation of the optimal model (MLP-WOA) with an ordinary model (MLP) showed that WOA improved the ability of ordinary ML for runoff simulation in the snow-covered catchment. The scatter plot of MLP-WOA showed that most of the data have fallen close to the best fit line.

**Table 11:** The results of the applied metrics on the calibration and validation phases of the MLP and coupled MLP-WOA models.

Model	Phase	*MAE*	*RMSE*	*NSE*	*r*	*KGE*
MLP	Training	5.33	9.61	0.68	0.82	0.75
	Testing	4.93	9.49	0.61	0.79	0.61
MLP-WOA	Training	5.14	9.07	0.71	0.85	0.78
	Testing	4.56	8.49	0.68	0.84	0.67

**Figure 10:**
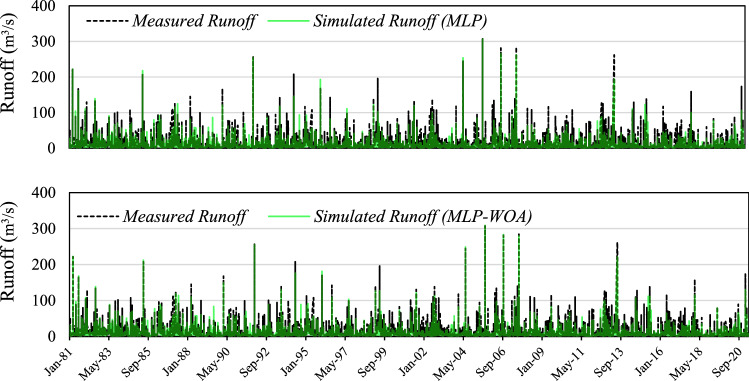
Time series plot related to the result of MLP and MLP-WOA models.

Hydrographs of the third phase are provided in Fig. 10 and it shows that the third phase is much more accurate than the second and first phases. According to Fig. 10, the time-series graph of MLP-WOA detected the maximum flow better than the other used strategies. It was successful in reproducing simulated hydrographs for both training and testing phases from 1981 to 2020.

### Runoff peak flow simulation analysis

**Figure 11:**
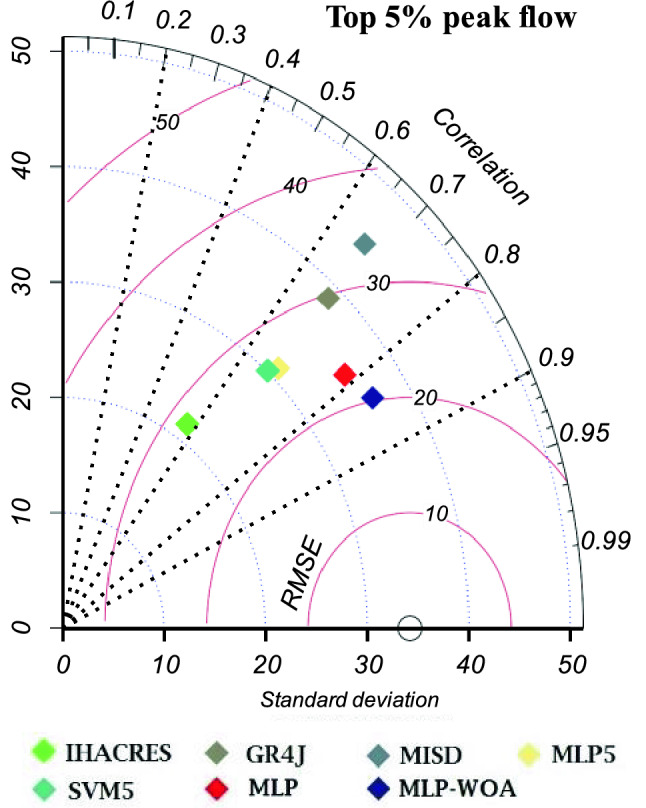
Taylor diagram of top 5% peak flow during the study period (1981–2021).

**Figure 12:**
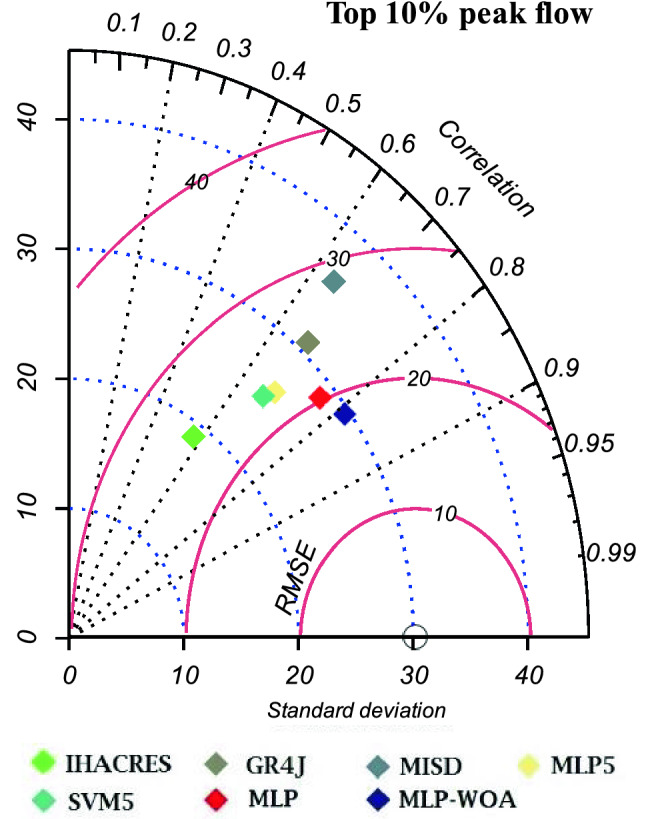
Taylor diagram of top 10% peak flow during the study period (1981–2021).

Maximum events of each model were analyzed by the Taylor diagram (Figs. 11 and 12). According to peak flow analysis for the top 5% and top 10% of peak flows, considered learning phases improved the ability of models for peak flow estimation. The ordinary hydrological models are located at the farthest point in both diagrams, and they have weak correlation and far standard deviation according to observed peak flow values. Second phase learning (adding meteorological variables by ML models) improved a little bit performance of peak flow estimation in all models. Then, the third learning phase (data-fusion: coupled hydrological models via ML models) dramatically improved the peak flow simulation. As it is shown in the diagram, the blue and red points are the results of the third learning phase, in which the blue point (MLP-WOA) has less error in peak flow simulation. It refers to the ability of hydrological models and ML models together in parallel conditions to have better runoff simulation in snow-covered basins. The statistical parameters given in Taylor diagrams (*RMSE, r,* and *SD*) for the top 5% and 10% of peak flow are listed in Table 12.

**Table 12:** *RMSE*, *r*, and *SD* of the top 5% and 10% of measured peak flow and simulated peak flow.

Metrics	IHACRES	GR4J	MISD	MLP5	SVM5	MLP	MLP-WOA
**Top 5% of peak flow**							
*RMSE*	43.11	41.3	41.88	34.95	36.47	32.46	29.14
*r*	0.57	0.67	0.67	0.69	0.67	0.78	0.84
*SD*	21.53	38.76	44.68	30.97	30.12	35.43	36.51
**Top 10% of peak flow**							
*RMSE*	32.6	32.27	33.25	27.24	28.14	25.4	23.15
*r*	0.58	0.68	0.64	0.69	0.67	0.77	0.81
*SD*	18.85	30.87	35.83	26.08	25.17	28.64	29.56

## Discussion

Since rainfall-runoff (RR) is a non-linear and complex hydrological phenomenon, a variety of approaches such as conceptual and empirical have been implemented for runoff estimation. Conceptual approaches which are known as physically-based or white-box models incorporate morphological and physical features of the problem and are quite useful for understanding the physics of the problem. However, in terms of accuracy, they may fail to generate satisfactory results. Alternatively, machine learning (ML) or black-box models have higher computational ability, while may fail in the physical justification of the problem. Therefore, the main research question of the study is to develop a methodology to merge the advantages of both aforementioned approaches to establish a robust-physically based model for runoff estimation. Three conceptual models as the IHACRES, GR4J, and MISD are developed in a snow-covered basin in Switzerland and then through using these models’ outcomes and a variety of hydro-meteorological parameters, the ML models of SVM, MLP, fusion MLP, and MLP-WOA are developed.

Results of conceptual models illustrate that the IHACRES, GR4J, and MISD models give almost the same results while GR4J provides slightly better results in contrast to the IHACRES and MISD models. Evaluation of the conceptual models’ performances in terms of computing the peak runoff values, IHACRES fails to an accurate estimation where it underestimates the peak values. Contrary, the MISD model overestimates several peak values. From a general point of view, conceptual models’ results are not satisfactory and it was the main motivation to couple these models with ML models for the Emme catchment. To overcome this issue, a variety of scenarios are defined to develop the IHACRES, GR4J, and MISD-based MLP and SVM models. For this purpose, five different scenarios consisting of precipitation, temperature, evapotranspiration, relative humidity, and snow depth are considered. The incorporation of hydro-meteorological variables into the models promotes the accuracy of the models developed in the first stage where the result of the IHACRES model with *RMSE* of 11.61 is improved to 9.43 and 9.79 in IHACRES-based MLP and SVM models, respectively. It shows almost 20% improvement in the IHACRES-based MLP model in contrast to the IHACRES model. This improvement is found at almost 14% for GR4J and MISD-based MLP models. The better performances of conceptual-based ML models can be linked not only to the robustness of the ML techniques but also to incorporating the variety of hydro-meteorological parameters of precipitation, temperature, evapotranspiration, relative humidity, and snow depth into the models.

In order to further improve the accuracy of the applied models, a fusion and robust ML model based on the WOA are implemented. For this aim, the best results which are obtained by the IHACRES-based MLP and SVM models are used as model inputs. The IHACRES-based MLP-WOA model with an *RMSE* of 8.49 m^3^/s improved the performance of the ordinary IHACRES model by a factor of almost 27%. It can be considered a satisfactory achievement for runoff estimation by applying coupled conceptual-ML hydrological models.

The GR4J, IHACRES, and MISD have been applied to various RR studies. For instance, Shin and Kim^5^ tried to improve the IHACRES and GR4J models by testing multiple component combinations and eventually achieved the *NSE*s ranging from 0.5 to o.8. Recently, the subject of increasing the hydrological model accuracy using ML models gained a huge interest. Tikhamarine et al.^23^ showed the superiority of using a Least Square Support Vector Machine (LSSVM) compared to the MLP coupled with the optimization models (PSO and HHO) in RR modeling with *NSE* values of 0.4 to o.8 however, they did not apply any hydrological model. In another work, Lees et al.^22^ applied the LSTM to four different conceptual models over the entire UK and achieved an average *NSE* of 0.7 to 0.8.

Esmaeili-Gisavandani et al.^54^ employed the Soil & Water Assessment Tool (SWAT), Hydrologiska Byråns Vattenbalansavdelning (HBV), IHACRES, Australian water balance mode (AWBM), and Soil Moisture Accounting (SMA) models for RR modeling in the Hablehroud basin (in Iran). They coupled outputs of hydrological models with a black-box model (GEP) and the result of the coupled model showed that the black-box model can improve the ability of the white-box model for RR modeling. Their coupled model accuracy was reported by *NSE* = 0.56 at the validation phase, while the coupled model of the current study was reported as *NSE* = 0.68 at the validation phase. Ahmadi et al.^53^ applied SWAT, IHACRES, and ANN in the Kan watershed (Iran). They reported *RMSE* equal to 3.3 (m^3^/s) and 3.7 (m^3^/s) for the calibration phase of the SWAT and IHACRES models, respectively, and also they reported *RMSE* equal to 2.2 (m^3^/s) for the testing phase of ANN model^53^. Their study was implemented in a semi-dry climate zone and the models reported have acceptable accuracy. However, due to the role of snow in cold regions, RR modeling in snow-covered areas is expected with more errors compared with dry and semi-dry regions. In some other research, the authors considered streamflow lag-times as input of black-box models for runoff modeling aims^55–58^ and they reached higher accuracies in runoff modeling. While the current study focused on the RR modeling concept by considering all inputs at the *t* (same) time and by conserving several meteorological variables as input of black-box models to have interpretation meaning for RR modeling. Previous researches confirm the result of the current study, for example, Ditthakit et al.^13^ used the black-box model to increase the efficiency of the white-box model in Thailand. This means that the method presented in this study can be expanded by other models and also can generalize the implementation of this method in other regions with different climates.

Several studies focused on the RR modeling over the Emme catchment and the surrounding areas. In terms of comparison of the model accuracy with the previous studies in the region, Antonetti et al.^59^ assessed the flash flood modeling between May to July 2016 using a chain of hydrological, meteorological, and process-based runoff generation modules and obtained 0.1 to 0.8 and 0.5 to 0.8 for the *NSE* and *KGE*, respectively. Sikorska-Senoner and Quilty^60^ achieved 16–29% improvements by applying various data-driven models to the conventional hydrological model for the streamflow simulation on Klein Emme catchment (a neighbor catchment to the Emme catchment). They recommended the use of extreme variant boosting and Random Forest models as they demonstrated the best performance.

Uncertainty in hydrological modeling has been a major challenge. The current study like other hydrological modeling studies was affected by two main uncertainties. (*I*) Uncertainty in models’ input: conceptual hydrological models showed a significant uncertainty based on models’ input. Affecting global warming (even only 1 °C) can have a significant effect on the results of the IHACRES, GR4J, and MISD models. By increasing the temperature, snow cover, and evapotranspiration, the amount of runoff can significantly change. However, the current study tried to reduce this uncertainty by applying meteorological variables as extra inputs besides the required inputs of conceptual models. (*II*) Uncertainty in hydrological models’ parameters can be considered as another limitation of the current study. The current study tried to use some of the famous optimization methods for calibration of conceptual hydrological models’ parameters, but still, the models are sensitive to any unexpected or extreme event in a new climate area. That means, a short-term heavy rainfall or a cold season can have a significant effect on the conceptual models’ calibration process, and the results of models can vary in different climates. Also, due to the role of snow (and glaciers) runoff modeling in snow-covered basins always has more complexity. Then, the current study selected a snow-cover basin for providing a solution for solving such a problem. The literature review proved that in basins without snow (less complexity) levels 1 and 2 of the current study could most probably lead to an acceptable accuracy for runoff modeling. The climatic zone, the scale of the basin, absence of snow, and data availability are some of the factors which could be mentioned for the complexity of the runoff modeling, then the current study recommended applying the current framework to different climate zones.

## Conclusions

In this study, three conceptual approaches of IHACRES, GR4J, and MISD are implemented for modeling the RR process in a snow-covered basin in Switzerland. The Two well-known ML techniques (the SVM and MLP) are coupled with the conceptual IHACRES, GR4J, and MISD models. It is found that the conceptual models’ accuracies are prompted by a factor of 14–19% in comparison to the ordinary conceptual models. Among conceptual-based ML models, the IHACRES-based MLP model gives better performance. Incorporating the hydro-meteorological variables of precipitation, temperature, evapotranspiration, relative humidity, and snow depth significantly improved the accuracy of developed models. An advanced ML model constructed through WOA has improved the performance of the MLP-WOA model by a factor of 27% in contrast to the conventional IHACRES model. Results of this study demonstrate that coupling conceptual and ML models can provide satisfactory outcomes in terms of accurate computation and physical justification of the problem. The developed methodology overcomes the basic deficiencies of the conceptual and ML methods where the former may fail to generate accurate results and the latter masked the physics of the problem. The coupled approach of merging the conceptual and ML models takes advantage of white-box models (e.g., considering the hydrological interpretation of the catchment) and black-box models (e.g., runoff modeling with explicit and implicit relationships between data that is out of the ability of white-box models) to construct a more robust and reliable model. Utilizing different calibration methods for overcoming the hydrological models’ parameters uncertainty is recommended as a future research direction. It is highly recommended to check the ability of the proposed method (three considered phases) under changing climate conditions. It is recommended to apply machine learning algorithms as feature selection tools for finding the most effective variables and overcoming models’ input uncertainty. In addition, three lumped models were considered in the current study and it is recommended to compare the results of the proposed method with the distributed hydrological models in the snow-covered basins as an extension of the current study.

## Data Availability

The datasets used and/or analyzed during the current study are available from the corresponding author on reasonable request.
